# Comparative transcriptome analysis of a long-time span two-step culture process reveals a potential mechanism for astaxanthin and biomass hyper-accumulation in *Haematococcus pluvialis* JNU35

**DOI:** 10.1186/s13068-019-1355-5

**Published:** 2019-01-28

**Authors:** Luodong Huang, Baoyan Gao, Manman Wu, Feifei Wang, Chengwu Zhang

**Affiliations:** Department of Ecology, Institute of Hydrobiology, College of Life Science and Technology, Jinan University, No.601 Huangpu Road, Tianhe District, Guangzhou, 510632 Guangdong People’s Republic of China

**Keywords:** *Haematococcus pluvialis*, Nitrogen starvation, Astaxanthin, Biomass concentration, Transcriptome, Metabolic pathway

## Abstract

**Background:**

Among all organisms tested, *Haematococcus pluvialis* can accumulate the highest levels of natural astaxanthin. Nitrogen starvation and high irradiance promote the accumulation of starch, lipid, and astaxanthin in *H*. *pluvialis*, yet their cell division is significantly retarded. Accordingly, adaptive regulatory mechanisms are very important and necessary to optimize the cultivation conditions enabling an increase in biomass; as well as promoting astaxanthin accumulation by *H*. *pluvialis.* To clarify the intrinsic mechanism of high-level astaxanthin and biomass accumulation in the newly isolated strain, *H. pluvialis* JNU35, nitrogen-sufficiency and nitrogen-depletion conditions were employed. Time-resolved comparative transcriptome analysis was also conducted by crossing the two-step culture process.

**Results:**

In the present study, we report the overall growth and physiological, biochemical, and transcriptomic characteristics of *H. pluvialis* JNU35 in response to nitrogen variation. From eight sampling time-points (2 days, 4 days, 8 days, 10 days, 12 days, 14 days, 16 days, and 20 days), 25,480 differentially expressed genes were found. These genes included the significantly responsive unigenes associated with photosynthesis, astaxanthin biosynthesis, and nitrogen metabolic pathways. The expressions of all key and rate-limiting genes involved in astaxanthin synthesis were significantly upregulated. The photosynthetic pathway was found to be attenuated, whereas the ferredoxin gene was upregulated, which might activate the cyclic electron-transport chain as compensation. Moreover, the expressions of genes related to nitrogen transport and assimilation were upregulated. The expressions of genes in the proteasome pathway were also upregulated. In contrast, the chloroplasts and nonessential proteins were gradually degraded, activating the specific ornithine–urea cycle pathway. These changes may promote the sustained accumulation of astaxanthin and biomass.

**Conclusions:**

To the best of our knowledge, this paper is the first to investigate the long-term differences of gene expression from two-step culture process in the astaxanthin producer, *H. pluvialis* JNU35. According to our results, β-carotene ketolase (*bkt1* and *bkt2*) serves as the key enzyme regulating astaxanthin accumulation in *H. pluvialis* JNU35. The cyclic electron-transport chain and novel nitrogen metabolic process were used adaptively as the regulatory mechanism compensating for different levels of stress. The in-depth study of these metabolic pathways and related key genes can reveal the underlying relationship between cell growth and astaxanthin accumulation in *H. pluvialis* JNU35.

**Electronic supplementary material:**

The online version of this article (10.1186/s13068-019-1355-5) contains supplementary material, which is available to authorized users.

## Background

*Haematococcus pluvialis* is a unicellular green alga that can hyper-accumulate astaxanthin under various stress conditions. Its typical astaxanthin content is 1.5–3.0% dry weight [1]. Under certain conditions, the astaxanthin content can be as high as 5–6% dry weight [2]. *H. pluvialis* can accumulate the highest content of natural astaxanthin reported to date [3]. However, due to the effects of genetic differences among various strains, many problems arise with the mass cultivation of *H. pluvialis*. These problems include cultures that are easily contaminated, or display slow growth rates and complex induction conditions. Thus, it is vital to optimize the cultivation conditions to increase biomass and promote astaxanthin accumulation of *H*. *pluvialis* at large-scale production [4–6]. However, the entire transcriptional regulatory mechanism of *H. pluvialis* is completely unknown, e.g., how *H*. *pluvialis* responds to stress and further accumulates astaxanthin, especially under nitrogen stress.

*Haematococcus pluvialis* has a complicated lifecycle, including a green vegetative cell phase and red immotile cyst cell stage where astaxanthin is accumulated [7]. At present, the industrial cultivation of *H. pluvialis* for commercial astaxanthin production is primarily achieved by a two-step mode. In the first mode, green vegetative cells are grown at low light with sufficient nutrition to obtain high cell density. In the second mode, when the density of green vegetative cells reaches its maximum, extreme stress conditions (e.g., high light and nutrient deficiency) could be used to induce astaxanthin accumulation [8, 9]. However, the improvement of astaxanthin yield remains limited by simply optimizing the culture conditions and culture modes. Thus, obtaining *H. pluvialis* strains with desirable genetic characteristics using screening and selection is key for efficient production of astaxanthin.

In our laboratory, a newly isolated strain of *H. pluvialis*, JNU35, showing a high capacity for biomass and astaxanthin accumulation, was obtained. In a two-step batch culture, *H. pluvialis* JNU35 biomass reached 4.21 g L^−1^ after 10 days of cultivation under nitrogen-sufficient condition. Subsequently, the culture was replaced with a nitrogen-free medium, and the final biomass reached 10.18 g L^−1^ after another 10 days of cultivation. With this high cell density and biomass concentration, the astaxanthin content reached 1.65% of the cell dry weight. In general, nitrogen starvation accelerated starch, lipid, and secondary metabolites’ accumulation in microalgae, yet their growth was significantly suppressed [10, 11]. Note that the net increase in biomass of *H. pluvialis* JNU35 was 5.97 g L^−1^ when the culture was replaced with nitrogen-free medium after 10 days’ growth, and the growth rate was even higher than that under nitrogen-sufficient (NS) condition. This process may be a special adaptive mechanism for growth and accumulation of astaxanthin. Accordingly, the regulatory mechanism of energy, carbon, and nitrogen allocations of *H. pluvialis* JNU35 under nitrogen starvation requires further study.

With the development of molecular biotechnology, the astaxanthin biosynthetic pathway in *H. pluvialis* has been clarified, and some key genes have been cloned successfully [12–15]. However, the genome of *H*. *pluvialis* has not yet been sequenced. Because there have been a few studies of the transcriptome and metabolic networks of *H*. *pluvialis* [16–19], the genes related to growth, astaxanthin, and energy metabolism remained unclear, especially the mechanism of adaptation and regulation under high light and nitrogen-starvation condition. In this study, the growth, photosynthetic characteristics, and biochemical components during the entire culture were determined. RNA-seq analysis was performed at eight time-points during a two-step culture. For the first time, the metabolic patterns of the vegetative cell growth stage and astaxanthin accumulation stage were compared dynamically, and the expression patterns of *H. pluvialis* JNU35 during the whole culture cycle were studied by transcriptome sequencing. By explaining the adaptive regulatory mechanism of *H*. *pluvialis* JNU35 under nitrogen starvation, these results should provide a perspective to solve the contradictory problems of biomass enhancement and astaxanthin accumulation of *H. pluvialis*. These findings also provide important guidance for the commercial production of *H. pluvialis.*

## Results and discussion

### Growth of *H. pluvialis* JNU35 under a two-step culture system

**Fig. 1:**
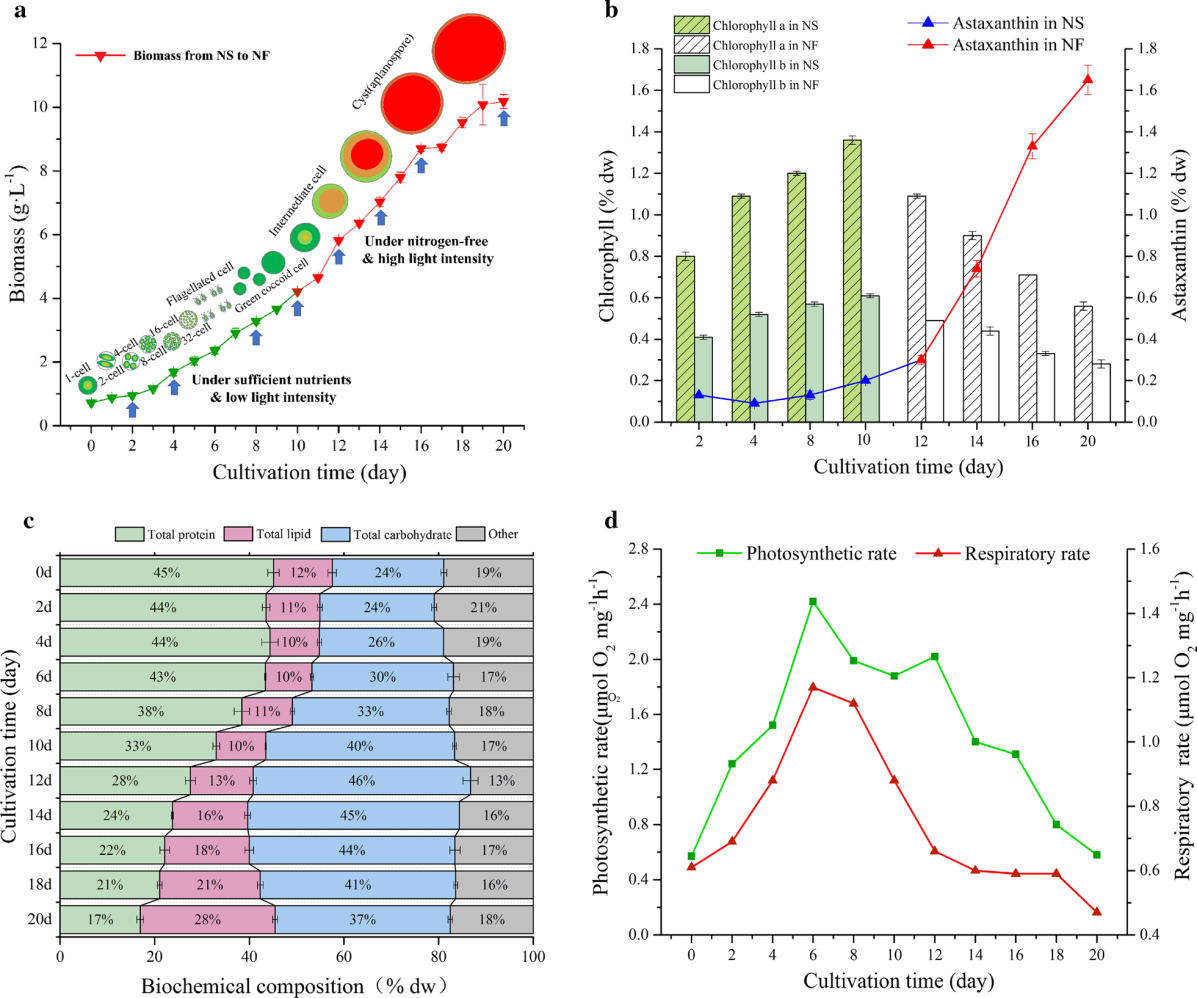
Physiological and biochemical characterizations of *H. pluvialis* JNU35 under the conditions from NS to NF. **a** Time-course changes in cell biomass concentration (the blue arrows represent sampling time-points for RNA sequencing). **b** Chlorophyll and astaxanthin content variations (% dw). **c** Changes in the biochemical composition. **d** Photosynthetic rate and respiratory rate

To analyze the growth of *H. pluvialis* JNU35 under a two-stage culture, *H. pluvialis* JNU35 was first cultured in nitrogen sufficient (NS) [9.0 mM (NH_2_)_2_CO] mBBM for 10 days and then transferred into a fresh nitrogen free (NF) (without nitrogen) mBBM for another 10 days. The growth patterns of *H*. *pluvialis* JNU35 cultivated from NS to NF were shown in Fig. 1a.

In the later stage of a two-step culture, cell division was blocked due to the lack of nitrogen, so each cell was exposed to relatively higher light intensity than NR stage, and biochemical compounds were rapidly accumulated after the compounds were transferred to the fresh nitrogen-free medium. It is noteworthy that the biomass of *H. pluvialis* JNU35 was 4.21 g L^−1^ on the 10th day (Fig. 1a). When the culture was replaced with the nitrogen-free medium, the biomass increased significantly under this double-stress condition (nitrogen starvation and high light). The final biomass concentration reached 10.18 g L^−1^ on the 20th day. The biomass showed a net increase of 5.97 g L^−1^ in the nitrogen-free stage (NF stage). Under these combined stresses, *H. pluvialis* JNU35 could maintain a high accumulation of biomass and showed the special response mechanism.

### Chlorophyll and astaxanthin variations of *H. pluvialis* JNU35 under the conditions from NS to NF

The pigment contents of *H*. *pluvialis* JNU35 under the conditions from NS to NF were determined, and the results are shown in Fig. 1b. With the extension of culture time, the contents of chlorophyll *a* and chlorophyll *b* increased gradually. These chlorophylls took up 1.36% and 0.61% of the dry weight on day 10, respectively. When the culture was under NF, the chlorophyll content decreased. Chlorophyll *a* and *b* only took up 0.56% and 0.28% of the dry weight on the 20th day, respectively. This result suggested that chlorophyll in *H*. *pluvialis* JNU35 degraded gradually, and the photosynthetic ability was affected by double-stress. Contrary to the change of chlorophyll content, there was little change in astaxanthin content in the 10 days under NS, and it was maintained at 0.2% of dry weight. When the cells were transferred into the NF, the astaxanthin content rapidly increased and reached 1.65% of the dry weight at the 20th day. According to these results, nitrogen depletion and high light could promote astaxanthin accumulation.

### Analysis of biochemical components of *H. pluvialis* JNU35 under the condition from NS to NF

The Fig. 1c shows the changes of total lipid, carbohydrate and protein contents of *H*. *pluvialis* JNU35 at different time phases under the condition from NS to NF. There was no significant difference in the lipid content under the NS stage, which was nearly 10% of dry weight. When the culture was replaced with NF medium, the total lipid content increased rapidly, taking up 28% of the dry weight on the 20th day. When the cultivation time was extended, the carbohydrate content increased first. The content of carbohydrate on day 12 took up 46% of the dry weight and then decreased to 37% on the 20th day. In late stage culture, the intermediate substrates and energy resulting from the degradation of storage compounds and carbohydrates were used for the metabolism of other essential substances and energy supply.

The content of protein, the component containing nitrogen in cells, remained stable in early period of NS stage (43–45%) (Fig. 1c). Subsequently, cellular protein was degraded for nitrogen consumption. The content comprised 33% of the dry weight on the 10th day and decreased to 17% on the 20th day of culture. This result was similar to *Chlamydomonas reinhardtii* in high light and low nitrogen stress, in which the protein of the cells degraded gradually and reallocated for nitrogen utilization [20].

### Photosynthetic characteristic analysis of *H. pluvialis* JNU35 under the conditions from NS to NF

The photosynthetic and respiratory rates of *H*. *pluvialis* JNU35 under conditions from NS to NF were measured with a Liquid-Phase Oxygen Measurement System (Fig. 1d). According to the results, both the photosynthetic and respiratory rates increased first and then decreased, and the highest values were 2.42 and 1.17 µmol O_2_ mg^−1^ h^−1^ achieved on the 6th day, respectively. The photosynthetic rate decreased gradually and reached 0.58 µmol O_2_ mg^−1^ h^−1^ on 20th day, a date close to the value of respiratory rate (0.47 µmol O_2_ mg^−1^ h^−1^). This result suggested that the photosynthetic capacity of *H*. *pluvialis* JNU35 decreased as the degree of stress. However, it was noteworthy that *H*. *pluvialis* JNU35 maintained a high photosynthetic rate even under double-stress. Previous studies had also found that the thylakoid membrane of *H. pluvialis* was gradually segmented and broken at the late stage of high light stress, while photosynthetic ability was decreased, only limited to a certain extent [21]. This result may indicate that the strain, *H. pluvialis* JNU35, had a special strategy to resist high light stress and maintained a certain level of photosynthesis.

### De novo transcriptome assembly, functional annotation, and RNA-Seq quantitation analysis of *H. pluvialis* JNU35

Due to the lack of genomic information for *H. pluvialis*, reference transcriptome data of *H*. *pluvialis* JNU35 was compiled. A reference transcriptome of 11.13 Gb was obtained. Using deep sequencing of mixed samples, enhanced transcript information was obtained and a complete gene set was provided with high sequencing depth and covering low-expression genes. This technique has also been applied to other studies to analyze the expression of several samples [22–24]. After obtaining high-quality clean-reads data, the clean reads were assembled. After assembling and eliminating redundancy, 63,132 unigenes with an N50 length of 1479 bp were obtained (Table 1). Subsequently, after assembling unigenes for functional annotation, a final 38,717 unigenes (61.33%) were annotated (Table 1). This high-quality reference transcriptome could be applied in the subsequent analysis of genes’ transcriptomic pattern.

**Table 1:** The *de novo* transcriptome assembly and annotation of *H. pluvialis* JNU35

Statistics	Results
Assembly	
Total number	63,132
Total length (bp)	55,541,141
Mean length (bp)	879
N50 (bp)	1479
GC (%)	59.61
Annotation	
Nr	34,759 (55.06%)
SwissProt	24,496 (38.80%)
Interpro	19,677 (31.17%)
KEGG	26,881 (42.58%)
GO	9497 (15.04%)
All annotated	38,717 (61.33%)

### Identification of differentially expressed genes (DEGs) of different time-points

To analyze the transcriptional response of *H*. *pluvialis* JNU35 under two-step culture conditions, eight time-point samples (2 days, 4 days, 8 days, 10 days, 12 days, 14 days, 16 days, and 20 days) were collected under the condition from NS to NF, Also, a transcriptome sample crossing the whole culture cycle was first selected (Fig. 1a). Time-resolved experiments were performed for the eight time-points and RNA-seq quantitation data were obtained from individual samples with an average of 53 Mb clean reads [Q20 was 92% (Additional file 1: Table S1)]. Mapped to the reference transcriptome, the average mapped reads rate was 87.75% (Additional file 1: Table S1), completely covering the low expression genes at each time-point.

**Fig. 2:**
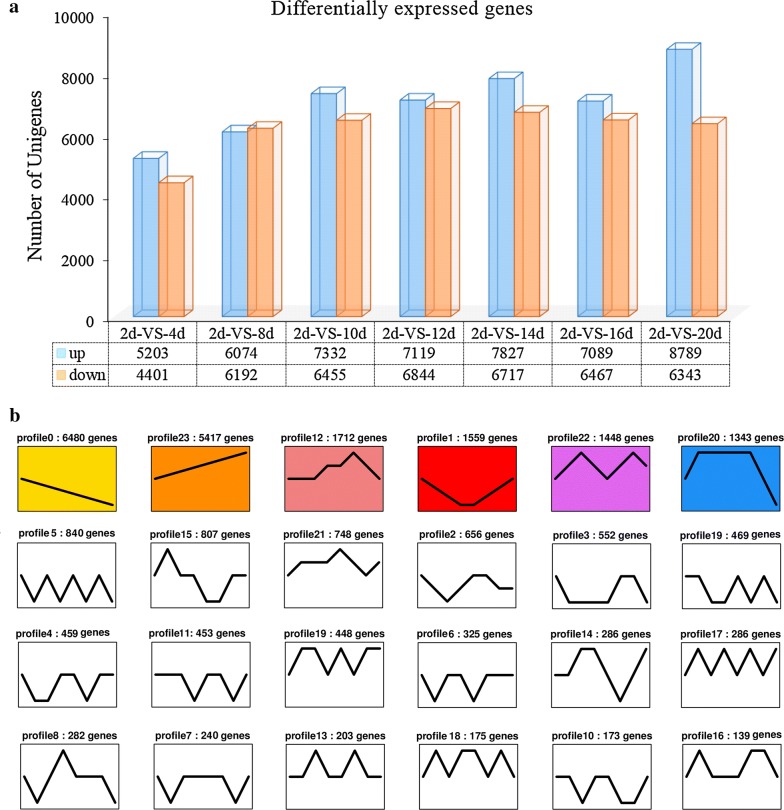
Screening of differentially expressed genes. **a** The number of DEGs (FDR ≤ 0.001 and fold-change D ≥ 2). **b** 24 expression profiles of DEGs

The expression of unigenes in each sample was calculated and normalized to FPKM. The DEGs between the experimental groups (4 days, 8 days, 10 days, 12 days, 14 days, 16 days, and 20 days) and the control group (2 days) were identified by multiple hypotheses testing based on the Poisson distribution with false discovery rate (FDR) setting of less than that of 0.001 and fold change ≥ 2 (log2 ratio ≥ 1) (Fig. 2a). There were a total of 25,480 DEGs at eight time-points compared to the control group. We note that in the culture during the later period of NF stage, more DEGs were found, suggesting that NF stage gene expression remained active. These DEGs must be correlated to the regulation of *H*. *pluvialis* JNU35 during the late period under double-stress conditions.

To further investigate the expression patterns and functions of DEGs contained in the eight time-points, the DEGs were clustered by STEM [25]. The total 25,480 DEGs were divided into 24 profiles (Fig. 2b). 17,959 (70.5%) of these unigenes were clustered into six profiles (*p*-value < 0.05), which included a downregulated trend for profile 0 (6480) and upregulated trend for profile 23 (5417), profile 12 (1712) and profile 20 (1343) representing an upregulation first and then a downregulation, profile 1 (1559) indicating a downregulation first and then an upregulation. Finally, profile 22 (1448) was volatile, fluctuating between upregulation (8 days and 16 days) and downregulation (12 days and 20 days).

### Gene Ontology (GO)-enrichment analysis of DEGs

**Fig. 3:**
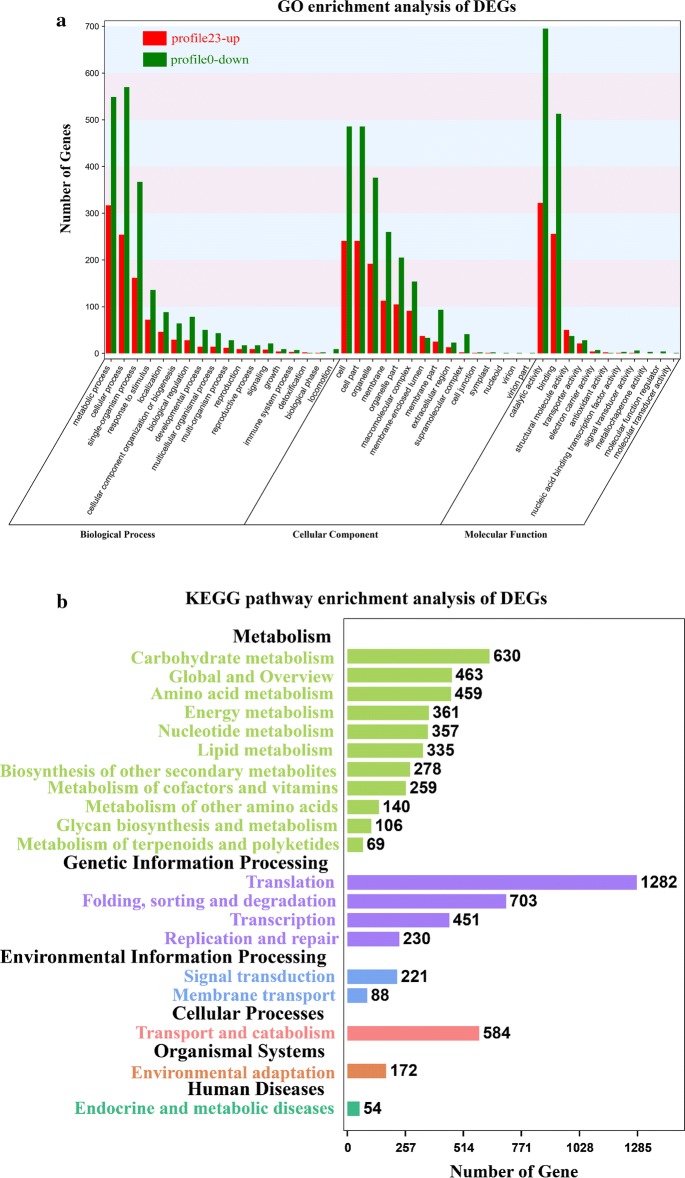
GO and KEGG annotation of DEGs. **a** GO classification of profile 23 and profile 0. **b** KEGG pathway enrichment analysis

Six significant profiles (*p*-value < 0.05) (profile 0, profile 1, profile 12, profile 20, profile 22, and profile 23 genes) were further classified by GO-enrichment analysis (Fig. 3a; Additional file 2: Table S2). A profile 0 (6480 genes) and profile 23 (5417 genes) contained the largest number of unigenes, which were classified into 2262 and 1521 GO terms, respectively. The unigenes of profile 0 and profile 23 were based on three major categories, namely biological process, cellular component, and molecular function, as shown in Fig. 3a. The biological process nodes were selected for the subsequent step of analysis.

In accordance with the GO biological process category, the isoprenoid biosynthetic process (GO:0008299), carotenoid biosynthetic process (GO:0016117), terpenoid biosynthetic process (GO:0016114), lipid biosynthetic process (GO:0008610), and acetyl-CoA carboxylase complex (GO:0009317) in profile 23 were upregulated (Fig. 3a; Additional file 2: Table S2), suggesting the accumulation of lipid and carotenoid in *H*. *pluvialis* JNU35 under the conditions from NS to NF.

Profile 0 represented a continuous downregulation pattern. The DEGs in this profile were primarily related to cell division and photosynthesis, which included photosynthesis (GO:0015979), light harvesting (GO:0009765), cellular amino acid metabolic process (GO:0006520), DNA replication (GO:0006260), tetrapyrrole biosynthetic process (GO:0033014), and regulation of cell cycle (GO:0051726) (Additional file 2: Table S2).

Furthermore, profile 12 and profile 20 represented the upregulation (stabilization) at first and the downregulation in the later culture stage. The DEGs of these were primarily related to the degradation of intracellular substances and energy metabolism, which included protein catabolic process (GO:0030163), organic substance catabolic process (GO:1901575), purine nucleoside triphosphate metabolic processing (GO:0009144), ATP generation from ADP (GO:0006757), and nucleosome assemblage (GO:0006334) (Additional file 2: Table S2).

### KEGG pathway enrichment analysis of DEGs

A total of 25,480 DEGs were analyzed for pathway enrichment in KEGG, and 22.6% (5746) of DEGs could be annotated. The metabolic pathways were significantly enriched in RNA transport, endocytosis, protein processing in endoplasmic reticulum, spliceosome, carbon metabolism ribosome, sucrose metabolism, fatty acid metabolism, and photosynthesis (Fig. 3b; Additional file 3: Table S3).

Six significant profiles also underwent KEGG enrichment analysis to identify metabolic or signal transduction pathways. A maximum number of DEGs were annotated in the RNA transport pathway. 225 genes were annotated to RNA transport in profile 0, whereas in profile 23, there were only 94 genes annotated to this pathway. In the all-culture cycle, the RNA of algal cells was transcribed and continuously transferred from the nucleus to the cytoplasm. Endocytosis, a pathway of cell nutrition absorption to take up extracellular nutrients into cells, contained 122 and 48 genes in profile 0 and profile 23, respectively. According to these data, exogenous nutrients decreased with the consumption of nutrients in culture medium. Hence, the expressions of genes related to endocytosis were downregulated, and cell nutrient absorption decreased. In profile 23, there were 19 (68% of DEGs) and 7 (29%) DEGs annotated in the terpenoid backbone biosynthesis and carotenoid biosynthesis pathways, respectively, associated with astaxanthin synthesis. These results revealed that many genes in these two pathways were upregulated to promote astaxanthin synthesis. The 41 (46%) DEGs of profile 0 and 17 (19%) DEGs of profile 23 were classified into the photosynthesis pathway, suggesting that genes related to photosynthesis were significantly suppressed. Furthermore, 36 DEGs in the proteasome pathway originated from profile 20, indicating that cells initiated the process of protein degradation to resist nitrogen-starvation stress.

### Reconstruction of the astaxanthin biosynthetic pathway in *H. pluvialis* JNU35

**Fig. 4:**
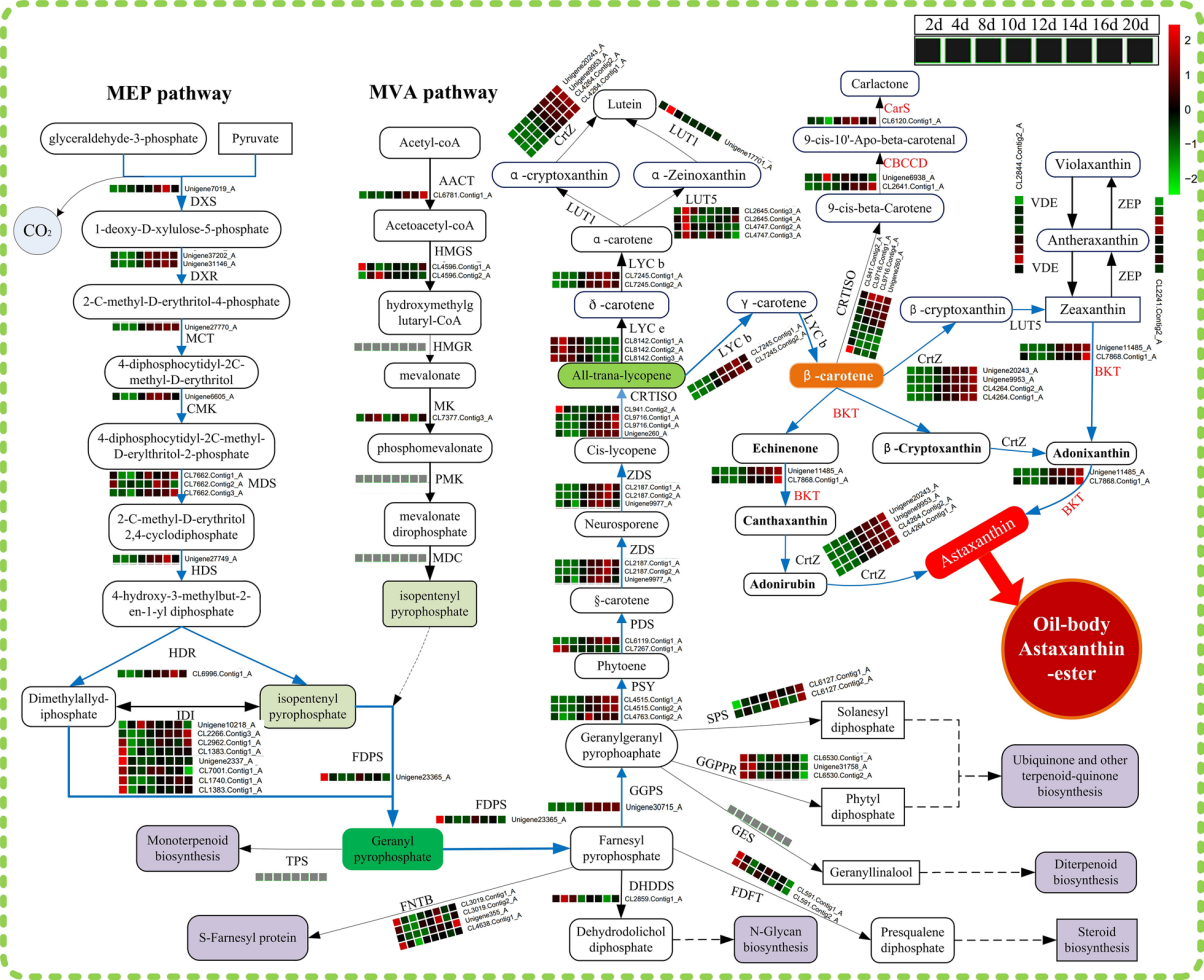
Schematic diagram of putative genes and their expression patterns in the biosynthetic pathway of astaxanthin in *H. pluvialis* JNU35. Genes’ expression patterns of each enzyme are represented by heat map (as FPKM). The upregulated genes in different time-points are indicated in red, downregulated ones are indicated in green, and no significant changes are shown in black. Each of the boxes shows the transcriptional regulation at the eight time-points. The full annotations of the corresponding genes are given in Additional file 4: Table S4

*Haematococcus pluvialis* is the main producer of natural astaxanthin [5, 26]. The astaxanthin synthetic pathway in algae can be divided into three steps [12]. The first step is to synthesize β-carotene precursor, isopentenyl pyrophosphate (IPP), via the MVA (3,5-dihydroxy-3-methyl-valonate) pathway in the cytoplasm or the MEP (2-C-methyl-d-erythritol-4-phosphate) pathway in the plastid [26, 27]. The second step is to synthesize β-carotene in chloroplasts, and the third step is to synthesize astaxanthin in the cytoplasm. Based on the transcriptomic data, the astaxanthin biosynthetic pathway of *H*. *pluvialis* JNU35 was reconstructed (Fig. 4; Additional file 4: Table S4).

In *H. pluvialis* JNU35, IPP can be synthesized via the MVA pathway or the MEP pathway. Eight different genes involved in the MEP pathway and six different genes involved in the MVA pathway were identified. The MEP pathway existed in the chloroplasts of eukaryotic algae and plants, starting with pyruvate and glyceraldehyde-3-phosphoric acid. This pathway is catalyzed by eight different enzymes [27–29]. According to the transcriptome analysis of *H*. *pluvialis* JNU35, the expression of the eight different enzyme genes in the MEP pathway are upregulated at different levels under the conditions from NS to NF as the culture time was extended, and most of these genes were upregulated more than twofold.

The first enzyme of the MEP pathway is 1-deoxy-d-xylulose-5-phosphatesynthase (DXS), catalyzed pyruvate, and glyceraldehyde-3-phosphate to form 1-deoxy-d-xylulose-5-phosphate. Subsequently, 1-deoxy-d-xylulose-5-phosphate was catalyzed by 1-deoxy-d-xylulose 5-phosphate reduction isomerase (DXR) to produce 2-C-methyl-d-erythritol-4-phosphate. These two enzymes are the rate-limiting enzymes in the MEP pathway. During the whole culture, the expression of one predicted DXS gene (unigene 7019A), and two predicted DXR genes (unigene 372020A and unigene 31146A) were upregulated gradually; the expression of unigene 7019A was upregulated by 3.8-fold on the 2nd day, and the unigene 372020A and unigene 31146A were upregulated by 1.75- and 2.2-fold on 16th day, respectively. Moreover, the other six key genes MCT (unigene 27770A), CMK (Unigene 6605A), MDS (CL7662. contig1A, CL7662. contig2A, and CL7662. contig3A), HDS (unigene 27749A), HDR (CL6996.contig1A) were all significantly upregulated by twofold. According to these results, the MEP pathway was significantly upregulated in the whole culture, especially in the NF stage, where it provides IPP for the subsequent synthesis of astaxanthin.

In addition to the MEP pathway, the MVA pathway may also exist in algae [30]. This pathway was first found in animals and yeast, the synthesis of triterpenoids and the sesquiterpenes in higher plants depends primarily on this pathway. It is completed in the cytoplasm [31]. The MVA pathway starts with acetyl-CoA, which is synthesized into IPP by six enzymatic reaction steps. According to the transcriptome annotated information of *H*. *pluvialis* JNU35, six different genes in the MVA pathway were identified. The expression level of the first enzyme gene in the MVA pathway was upregulated continuously during the whole culture time. In addition, the expressions of genes HMGSN (CL4596.contig2A) and MK (CL7377.contig3A) were upregulated by various degrees on the 2nd, 4th, and 8th days. However, no expression changes were detected between conditions in the other three identified genes (HMGRN, PMK, and MDC). This indicated that these genes did not respond to nitrogen starvation under the conditions from NS to NF. The expressions of genes involved in the MVP pathway were low (Fig. 4; Additional file 4: Table S4), but the expressions of the eight genes in the MEP pathway were significantly upregulation, especially in the late stage of NF stage. This outcome suggested that the MEP pathway was the main pathway to provide IPP for carotenoid synthesis in *H. pluvialis* JNU35.

IPP is an important intermediate of the MEP and MVA pathways. Under catalysis by isopentenyl-diphosphate delta-isomerase (IDI), IPP can be isomerized into dimethylallyl-diphosphate (DMAPP). It was found that the expression level of IDI can directly affect the metabolic flow of precursor substances in the carotenoid synthesis pathway and has a regulatory effect on the downstream metabolic pathway [32]. Two different types of IDI genes were found in *H. pluvialis* JNU35, which were identified in the cytoplasm and chloroplast, respectively. Ten unigenes of *H*. *pluvialis* JNU35 were annotated as IDIs. By means of the subcellular localization analysis software ProtComp9.0 (http://www.softberry.com), four predicted IDIs (CL1383.contig1A, CL2266.contig3A, CL2962.contig1A, and unigene10218A) might locate in chloroplasts with high levels of expression. The expression of CL2266.contig3A was still upregulated by twofold on the 20th day. In addition, the other three predicted IDIs (CL1740.contig1A, CL7001.contig1A, and unigene2337A) might locate in the cytoplasm, and downregulated at NF stage. These results showed that the IDI in chloroplast was related to astaxanthin synthesis in NF stage. Combined with the gene expressions of MEP and MVA pathway, IPP is synthesized predominantly by the MEP pathway in *H*. *pluvialis* JNU35.

The second step of astaxanthin biosynthesis is β-carotene synthesis. IPP and DMAPP are catalyzed by farnesyl diphosphate synthase (FDPS) to generate the geranyl pyrophosphate, which is then catalyzed by geranylgeranyl diethylene synthase (GGPS) to produce the geranylgeranyl pyrophosphate. Finally, geranylgeranyl pyrophosphate is catalyzed by phytoene synthase (PSY) to form phytoene to be incorporated into the β-carotene synthesis pathway. One of the four predicted GGPS genes (unigene 30715A) recorded 3.2-fold of upregulation on the 20th day. Two of the four predicted PSY unigenes (CL4515.contig1A and CL4515.contig2A) recorded 9.3- and 9.2-fold upregulations, respectively, on the 20th day.

From phytoene, all the key genes involved in the synthesis of astaxanthin were significantly upregulated in the NF stage of high light and low nitrogen stress (Fig. 4; Additional file 4: Table S4), including phytoene desaturase (PDS) (CL6119. contig1_A, 7.2-fold upregulated on the 16th day), δ-carotene desaturase (ZDS) (CL2187.contig1_A, CL2187. contig2_A, and unigene 9977_A; 5.3-, 5.2- and 1.5-fold upregulated, respectively, on the 16th day), carotene isomerase (CRTISO) (CL9716. contig1A, CL9716. contig2A, and unigene 260A; 1.8-, 3- and 1.7-fold upregulated, respectively, on the 20th day), lycopene beta cyclase (LYCb) (CL7245. contig1A and CL7245. contig 2A; 8.7- and 11-fold upregulated, respectively, on the 16th day), β-carotene 3-hydroxylase (CrtZ) (unigene 20243A, unigene 9953A, CL4264.Contig1A, and CL4264. contig 2A; 40-, 425-, 244-, and 26-fold upregulated, respectively, on the 20th day) (Fig. 4; Additional file 4: Table S4).

It is noteworthy that CrtZ and β-carotene ketolase (BKT) catalyzes the last step in the synthesis of astaxanthin in *H. pluvialis* [26, 33]. BKT is the only enzyme that exclusively participated in the astaxanthin synthesis pathway, and is thus directly related to the synthesis of astaxanthin [34]. There are three types of BKT genes in *H. pluvialis*, *bkt1*, *bkt2*, and *bkt3* [35–37]. The reported sequences of bkt1, bkt2, and bkt3 (GenBank accession number: GU143688; GU143689; GU143690) were downloaded, and through blast alignment, the *H*. *pluvialis* JNU35 transcripts of unigene11485_A was found to be 98%, similar to *bkt1*. The similarity of CL7868. contig1_A with *bkt2* or *bkt3* reached 98%. Also, the gene sequences of *bkt3* and *bkt2* appear to be highly similar, and only 22 bases different [37]. However, the enzymes encoded by *bkt1*, *bkt2*, and *bkt3* can catalyze the conversion of β-carotene to echinenone and canthaxanthin [34]. The transcriptome of *H*. *pluvialis* JNU35 showed that the expressions of two different types of BKT genes, unigene11485_A and CL7868.contig1_A, were significantly upregulated under NF stage. They were 23- and 2.5-fold upregulation on the 20th day compared to the expressions on the 2nd day, respectively. This result suggested that *bkt1* (unigene11485_A) was more responsive to astaxanthin synthesis. Similarly, the efficiency of *bkt1* in *C. zofingiensis*, an alga that also accumulates astaxanthin, was found to be higher than that of *bkt2* [37]. Therefore, we speculate that the enzyme that the *bkt1* gene encodes is the main contributor to astaxanthin synthesis.

With the upregulation of key genes in astaxanthin synthesis, some bypass pathways and genes that synthesize competitive intermediates were suppressed. Their expression was continuously downregulation from the early or middle NS stage (Fig. 4; Additional file 4: Table S4). For example, geranyl pyrophosphate is also the synthetic precursor of monoterpenoid. Geranyl pyrophosphate is catalyzed by linalool synthase (TPS) to enter monoterpenoid biosynthesis pathway. The two predicted TPS unigenes of *H*. *pluvialis* JNU35 were not expressed significantly which indicated that the synthesis of monoterpenoids was suppressed. Similarly, farnesyl pyrophosphate is catalyzed by ditransapoly cis-polyprenyl diphosphate synthase (DHDDS), farnesyl-diphosphate farnesyltransferase (FDFT), and protein farnesyltransferase β subunit (FNTs) to proceed to *N*-glycan biosynthesis, steroid biosynthesis, and *S*-farnesyl protein, respectively. These three genes were upregulated at the early NS stage and downregulated at the middle NS stage and NF stage. The enzymes that compete with PSY for the geranylgeranyl pyrophosphate substrate include geranyllinalool synthase (GES) and geranylgeranyl diphosphate reductase (GGPPR). Their expressions were upregulated in early NS stage and downregulated in NF stage. This result indicated that the expressions of genes related to the synthesis of important precursors of terpenoid, chlorophyll, and tocopherol were downregulated. These substances play a specific role in cell growth and metabolism, and the syntheses of these intermediates were suppressed during astaxanthin synthesis. However, the expressions of all-trans-nonaprenyl-diphosphate synthases (SPS) (CL6127. contig1_A and CL6127.contig2_A) were upregulated (by 1.6- and 1.4-fold, respectively) on the 20th day. SPS catalyze geranylgeranyl pyrophosphate to yield synthesize plastoquinol A. It has been reported that plastoquinol A could be synthesized by algal cells under nitrogen stress to remove singlet oxygen [38]. The upregulation of these genes may be a response strategy to protect the PS II in *H*. *pluvialis* JNU35 under double-stress (high light and low nitrogen conditions) (Fig. 4; Additional file 4: Table S4).

Lycopene β-cyclase (LYC b) is a key enzyme in astaxanthin synthesis. It catalyzes all-trans-lycopene to produce γ-carotene into β-carotene pathways. But all-trans-lycopene could be also catalyzed by lycopene ε-cyclase (LYC e) and LYC *b* to generate α-carotene, which then produces lutein through two intermediates of α-cryptoxanthin and α-zeinoxanthin. Moreover, the lutein synthesis pathway can compete with astaxanthin synthesis for LYC *b* and reduce astaxanthin synthesis. According to the transcriptome analysis of *H*. *pluvialis* JNU35, the three annotated LYC e genes, CL8142.contig1_A, CL8142.contig2_A, and CL8142.contig3_A, were upregulated at the early NS stage and then downregulated. But they were not expressed at the later NF stage, and downregulated by more than 100-fold on the 20th day (Fig. 4; Additional file 4: Table S4). Therefore, the inhibition of a competitive pigment synthesis pathway was also a reason why *H*. *pluvialis* JNU35 could enrich astaxanthin in large quantities.

One of the astaxanthin synthetic pathways requires the production of adonixanthin via zeaxanthin. However, zeaxanthin could also be catalyzed by zeaxanthin epoxidase (ZEP) to produce antheraxanthin and violaxanthin. This pathway could be catalyzed reversibly by violaxanthin de-epoxidase (VDE) to generate zeaxanthin. This cycle was known as the violaxanthin cycle [39]. Prior research had suggested that the violaxanthin cycle is responsive to high light, and the cycle can dissipate excess light energy captured by chlorophyll molecules, thereby protecting chloroplasts from light damage. This finding was very significant for maintaining normal photosynthesis in cells [40]. The transcriptome analysis of *H*. *pluvialis* JNU35 showed that both ZEP (CL2241. contig2A) and VDE (CL2844. contig2_A) were upregulated at the early NS stage with the increase in light intensity. They might play a role in light protection to some extent. However, with the increase in stress, both genes were downregulated at the NF stage. This indicated that the violaxanthin cycle had a limited photoprotective effect [40]. Therefore, *H*. *pluvialis* JNU35 algal cells initiated another mechanism of astaxanthins which were used as shading agents and antioxidants to protect the light system [41, 42]. Astaxanthin biosynthesis was activated under high light and nitrogen-starvation conditions to protect cells from damage [3]. It upregulated all the genes associated with the astaxanthin biosynthesis pathway and inhibited those of competing pathways which promoted the rapid accumulation of astaxanthin (Fig. 1b and Fig. 4).

### Photosynthetic response of *H. pluvialis* JNU35 under the conditions from NS to NF

Photosynthesis is a complex metabolic process that includes light absorption, energy conversion, electron transfer, ATP synthesis, and CO_2_ fixation. It is considered the main energy source of photoautotrophic algae and provides ATP and NADPH for algal cell growth and metabolism [43]. *H. pluvialis* could hypersynthesize astaxanthin, which may be an adaptation to withstand strong light stress and to protect the light system from damage [3], but astaxanthin synthesis consumed large amounts of ATP and NADPH. With the extension of cultivation time, the photosynthetic rate of *H*. *pluvialis* JNU35 decreased gradually (Fig. 1d). Hence, *H*. *pluvialis* JNU35 cells may have other energy-generation strategies to accumulate astaxanthin. For photosynthesis, *H. pluvialis* requires a unique strategy to address high light intensity stress and to supply energy for growth and astaxanthin synthesis [21].

**Fig. 5:**
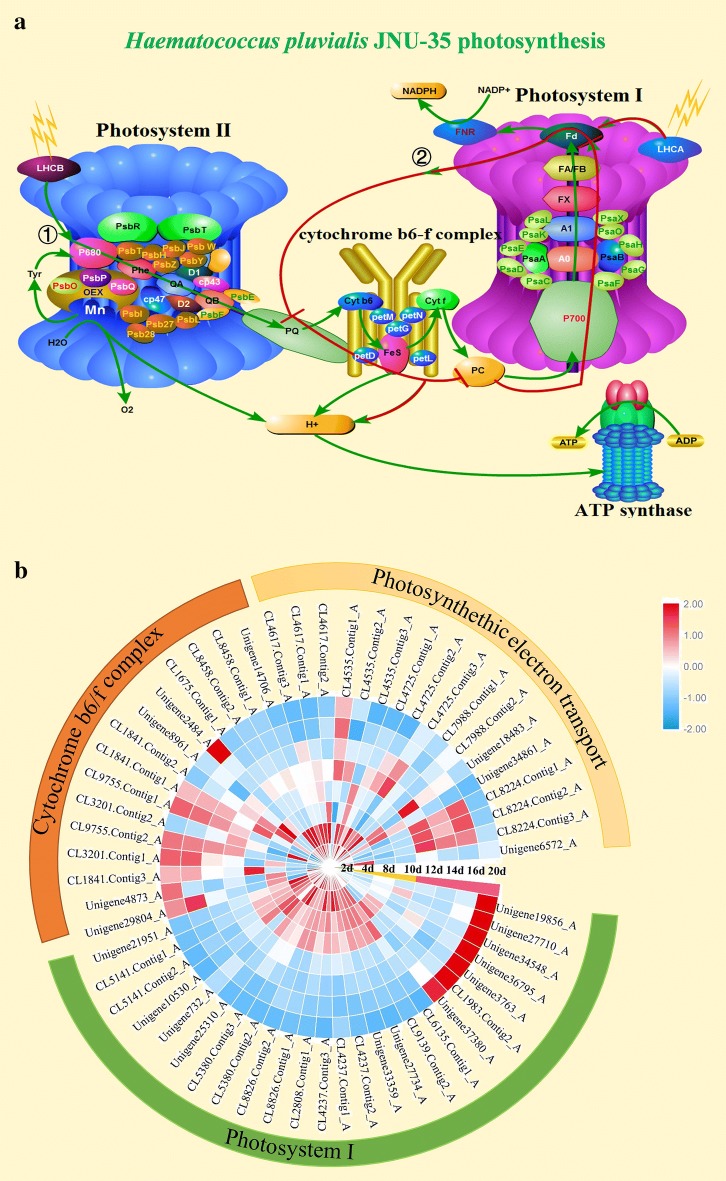
Photosynthetic pathway in *H. pluvialis* JNU35. **a** Schematic diagram of putative photosynthetic pathway, ①Green line: Z-type electron-transport chain; ②Red line: cyclic electron-transport chain. **b** Changes in expression patterns of gene involved in cyclic electron-transport chain

The photoreaction is primarily located in the thylakoid membranes and consists of four protein complexes, photosystem I, photosystem II, cytochrome b6-f complex, and ATPase (Fig. 5a). Under high light and nitrogen-starvation conditions, the photosynthetic rate of *H*. *pluvialis* JNU35 was decreased gradually, and the photosynthetic ability was significantly affected (Fig. 1d). The light-harvesting complexes (LHCs), belonging to the photosynthesis-antenna proteins, were the first proteins affected and could capture light energy quickly. Then, the captured light energy could be transferred into the reaction center of photosynthesis system [43]. PSII and PSI have their own the light-harvesting complexes, LHCB and LHCA, respectively. The LHCB of PSII is the most abundant membrane protein in algal cells [44]. From the transcriptome data of *H*. *pluvialis* JNU35, only three LHCB genes, LHCB1 (CL5994.contig1_A), LHCB3 (CL5994.contig2_A), and LHCB7 (unigene6939_A) were upregulated slightly. Accordingly, in PSII, the expressions of two central reactive proteins, D1 and D2, and the associated proteins CP43/CP47, which were antenna proteins of chlorophyll, were downregulated at the NF stage. The oxygen-evolving complex (OEX) contained three subunits, psbO, psbP, and psbQ. The expressions of three predicted psbO unigenes were downregulated under the NF stage, and 11 predicted psbP genes were downregulated gradually with extended culture time. But two predicted psbQs, CL1954.contig1_A and unigene12973_A, were upregulated. On the 20th day, their expressions were higher than that on the 2nd day (by 1.8- and 1.44-fold, respectively). The two peripheral proteins next to the OEX were psbR and psbT. The expressions of two predicted psbR (CL9335.contig3_A and CL9335.contig4_A) were upregulated and remained at 9.8- and 5.6-folds higher, respectively, on the 16th day. All the 15 predicted psbS genes showed an upregulation response pattern, which suggested that these genes might provide protection for the core of PSII to some extent. In addition, other protein subunits and peripheral proteins in PSII, such as psbE, psbF, psbW, psbY, psbH, psbJ, psbI, psbZ, psb27, and psb28, all showed varying degrees of downregulation (Additional file 5: Table S5). This indicated that the structure and activity of PSII were damaged, but they still maintained a certain photosynthetic activity. This result also coincided with the change of photosynthetic oxygen evolution as shown Fig. 1d.

The PSI reaction center contained 16 subunits [45]. The expressions of two core reaction center proteins, psaA and psaB, which bind chlorophyll and carotenoid for electron transport were low and stable. On the 20th day, the expressions of *psaA* and *psaB* were upregulated. However, the expressions of *psaD*, *psaE*, *psaF*, *psaG*, *psaH*, *psaI*, *psaK*, *psaL*, and *psaO* were downregulated, and their relative expression levels were maintained at a high level in the NF stage (Additional file 5: Table S5), suggesting that PSI retained relatively high activity in the NF stage of high light and nitrogen deficiency (Fig. 5b). It may play a certain role in the energy metabolism of cells in response to stress.

The cytochrome b6-f complex (*Cyt* *b6f*) is the rate-limiting step in the electron-transport process of photosynthesis. There are three electron-transfer subunits, petA, petB, and petC (Fig. 5a). Among them, petC is a 2Fe-2S iron-sulfur protein. The expressions of two predicted petC unigenes, CL9755.contig2_A and CL9755.contig1_A, were 20- and 30-fold upregulated on the 20th day, respectively. This indicated that many electrons were still transferred to plastocyanin (PC) at the NF stage. As an electron transporter, plastocyanin could flexibly accept the electrons transferred from *Cyt b6f*, and two predicted plastocyanin unigenes (CL4617.contig1_A and CL4617.contig3A) were downregulated gradually, but they still maintained a high expression level until the NF stage. This result showed that the photosynthetic system had decayed, but they still maintained the transfer of electrons to PSI in the whole culture cycle. Similarly, for ferredoxin (Fd), the last electron vector at the end of the electron transfer, 10 unigenes were predicted ferredoxin, and three of them were labeled in the chloroplasts (CL4725. contig1_A, CL4725. contig2_A, and CL4725. contig2_A). Their expressions were downregulated, but maintained at high expression levels at the NF stage. CL4725.contig3_A was still upregulated by 1.2-fold on the 16th day, indicating that the electron transfer in NADP+ reductase was increased at the NF stage, and then NADP^+^ was reduced to NADPH by ferredoxin-NADP+ reductase (FNR). Three predicted FNR unigenes (CL8224.contig1_A, CL8224.contig2_A, and CL8224. contig3_A) continued to maintain higher levels of expression, and no significant downregulation was observed at the NF stage (Fig. 5b). Furthermore, the 9 F-type H^+^-transporting ATPase subunits also maintained high expression levels. These photosynthetic electron-transporting genes’ expression patterns showed that the photosynthetic system of *H*. *pluvialis* JNU35 was attenuated under the NS to NF conditions (Fig. 5b), but maintained electron-transport activity until the NF stage. To analyze the strategies of energy metabolism under the NS and NF stages, the concentration of NADPH was measured, where from NS to NF, the content of NADPH considerably decreased (Table 2). These results pointed to the clear change in the maintenance of NADPH under NF stage and indicated that the photosynthetic system of *H*. *pluvialis* JNU35 maintained electron transport and produced NADPH and ATP for the growth of the cells and the accumulation of astaxanthin in the NF stage (e.g., high light and nitrogen deficiency).

**Table 2:** The NADPH levels of *H. pluvialis* JNU35

Cultivation time	NADPH concentration (µM g^−1^)
NS	
2 days	39.60 ± 1.42
4 days	55.94 ± 0.71
8 days	32.85 ± 0.07
10 days	13.51 ± 0.11
NF	
12 days	11.37 ± 0.16
14 days	8.05 ± 0.33
16 days	7.30 ± 0.03
20 days	5.99 ± 0.10

It was, however, still the case that the photosynthetic system was attenuated under the double-stress from high light and nitrogen starvation. The four protein complexes on thylakoid membrane (PSI, PSII, *Cyt b6f*, and ATPase) were also affected. In *H*. *pluvialis* JNU35, the normal Z-type electron-transfer chain (Fig. 5a ①-green line) and the synthesis rate of NADPH and ATP were affected to some extent. However, the algal biomass continued to increase to 5.97 g L^−1^ from the 10th to 20th days (NF stage, Fig. 1a). The content of astaxanthin, which required energy, was also increased by eight times (Fig. 1b). Perhaps there was an approach in the algae which was different from the Z-type electron-transfer chain to supply energy.

Several studies have shown that the activity of PS II was damaged by drought and other stresses, and the cyclic electron transport that contained PSI only was usually activated to generate energy [46]. In algae, the circular electron-transport flow is driven by a hyper-complex composed of PSI, cytochrome b6-f complex, and ferredoxin reductase (Fig. 5a ②-red line). Electron flows were passed through Fd, PC, *Cyt b6f*, and returned to PSI to form a cyclic electron flow. This was characterized by the lack of H_2_O oxidation and NADPH formation; however, H^+^ could be transported across the membrane to produce ATP [46].

Ferredoxin was the last common substance of all electron-transporting pathways in chloroplasts. FNR in the Z-type electron-transport chain competed with cyclic electron transport for ferredoxin (Fig. 5a ②-red line). Therefore, the ferredoxin content affected the operation of cyclic electron transport and controlled the ratio of two-electron transfer. The cyclic electron-transport chain was enhanced when the ferredoxin was overexpressed [47]. It is worth noting that in *H*. *pluvialis* JNU35, three chloroplast-located ferredoxin genes still maintained high levels of expression after PSII damage and were even upregulated at the NF stage (Fig. 5b; Additional file 5: Table S5). At the same time, the expressions of genes related to maintaining the cyclic electron-transport chain hypercomplex were significantly upregulated. We thus speculated that *H*. *pluvialis* JNU35 initiated the cyclic electron-transport chain pathway under stress. This produced NADPH and ATP in the NF stage to promote the cell growth and astaxanthin accumulation through the interaction of the Z-type and cyclic electron-transport chains (Fig. 5).

### Competitive absorption and metabolism of nitrogen in *H. pluvialis* JNU35 under nitrogen starvation

Nitrogen is the second major nutrient element in microalgae and could account for 1–14% of algae biomass [48]. Nitrogen is involved in the synthesis of many bioactive substances in microalgae. The three most common nitrogen sources used in microalgae are nitrate, ammonium, and organic nitrogen, such as urea. NO_3_^−^ is transported into the cell by nitrate/nitrite transporter (NRT) and then catalyzed by nitrate reductase (NR) to produce NO_2_^−^. The NO_2_^−^ is then transported to chloroplast and reduced to NH_4_^+^ by ferredoxin-nitrite reductase (NIR). However, extracellular urea is transported into the cell via the urea-active transporter (UAT) and then catalyzed by urease to form NH_4_^+^. NH_4_^+^ is transported into cells through the ammonium transporters (ATMs). Finally, the absorbed nitrogen sources enter the glutamine or glutamic acid synthesis pathway in the form of NH_4_^+^ to synthesize organic nitrogen compounds.

**Fig. 6:**
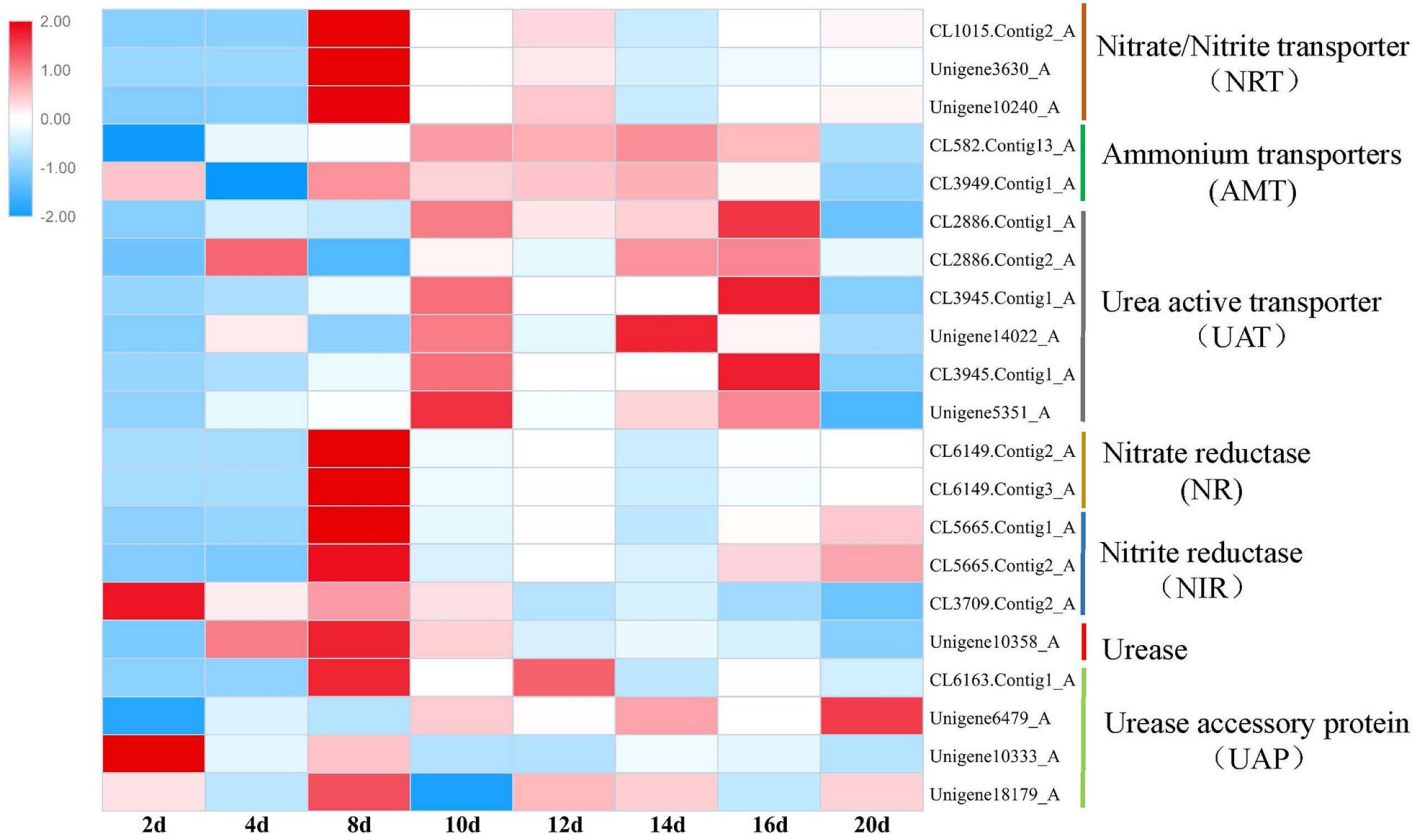
Heat map analysis of nitrogen absorption- and metabolism-related gene expression patterns

In this study, urea served as a nitrogen source. The urea in the culture medium was gradually consumed after 10 days (NS stage). Five predicted UAT and urease (unigene 10358_A) had relatively stable expression. On the 8th day of culture, there were slight upregulations, but no significant difference on the 20th day (Fig. 6; Additional file 6: Table S6). The expressions of four predicted urease accessory proteins (UAP) were also relatively stable and showed no significant differences during the whole culture under the conditions from NR to NF.

In contrast, the expression of three predicted NRT (CL1015.contig2_A, unigene3630_A, and unigene10240_A) were 66-, 77-, and 100-fold of upregulated on the 8th day, respectively. The two predicted NRs also showed the same expression patterns with the response of NRT to nitrogen deficiency. However, two predicted ATMs showed relatively stable expression patterns, and no significant downregulation was observed in the NF stage (Fig. 6). Under the NF stage of nitrogen starvation, algae cells rapidly induced the expressions of genes, which was related to nitrogen transport and assimilation to increase the absorption of exogenous nitrogen. In contrast, nitrogen transport and assimilation genes were expressed stably when nitrogen was sufficient. But it was hard to imagine that the expressions of NRT, NR, and AMT genes were maintained under the conditions of the absence of nitrate and ammonium. It was possible that algal cells would prepare to absorb nitrogen under long-term nitrogen starvation. Thus, when nitrogen occurred in the cultivation environment, it could be absorbed by the cell quickly and used to restore the cell’s growth.

### Endogenous nitrogen utilization of *H. pluvialis* JNU35 was activated under nitrogen starvation

When nitrogen is absorbed into algal cells, it is usually used in the form of NH_4_^+^, and then synthesized into nitrogen-containing compounds by the catalysis of glutamate dehydrogenase (GDH) and glutamine synthetase (GS). The expression of one of the two predicted GDHs, CL3883.contig1_A was upregulated, while the other, unigene23392_A, was gradually downregulated. These two gene products were likely located in mitochondria and chloroplasts, respectively. Seven of the nine predicted GSs were located in the cytoplasm, and their expressions were upregulated, while the expressions of other two genes (CL5112.contig1_A and CL5112.contig2_A) located in chloroplast were downregulated gradually (Additional file 6: Table S6). The chloroplast was degraded under nitrogen starvation, at the site where cellular metabolism was transferred from chloroplasts into the cytoplasm.

We note that *H*. *pluvialis* JNU35 had the associated genes of the ornithine-urea cycle pathway, which was also reported in diatoms [49]. The expressions of carbamoyl-phosphate synthetase (CPS), ornithine carbamoyl transferase (OTC), arginine succinate synthase (ASS), and arginine succinate lyase (ASL) continued to be upregulated during the under condition from NS to NF (Additional file 6: Table S6). However, the transcriptome data of *H*. *pluvialis* JNU35 lacked the last arginase gene of the urea cycle. The arginase catalyzed arginine to produce urea and new ornithine, further ensuring the reuse of urea. To date, there has been no report on the complete urea cycle in green algae and plants [49, 50]. However, other genes in the urea cycle pathway were significantly expressed, and the loss of arginase may reveal that there is a special ornithine pathway in *H*. *pluvialis* JNU35 and other green algae. They may recycle the nitrogen from the breakdown proteins via catabolic pathways in cells. The protein content of *H*. *pluvialis* JNU35 was decreased gradually when the culture time was extended, and the relative protein content on the 20th day was reduced by 50% (Fig. 1c). In response to the change in expression patterns of *GDH*, *GS*, the proteasome pathway-related genes, and endocytosis pathway-related genes, it was speculated that *H*. *pluvialis* JNU35 could use its own endogenous nitrogen under NS stress, wherein some endogenous nonessential nitrogenous substances were degraded to maintain essential amino acid synthesis [51].

### Verification of DEGs’ expression patterns through qPCR analysis

To verify transcriptome data, eight genes (*DXS*, *HDR*, *FDPS*, *GGPS*, *PSY*, *CrtZ*, *BKT1*, and *BKT2*) were employed for qPCR analysis. These genes were associated with MEP and carotenoid synthesis pathways, as well as key genes (*CrtZ*, *BKT1*, and *BKT2*) of astaxanthin synthesis (Additional file 7: Figure S1). According to the results, the expression patterns of most genes were consistent with transcriptome date, suggesting that transcriptome data were reliable.

## Conclusion

To our best knowledge, this study is the first to analyze the overall growth and physiological, biochemical, and transcriptomic performance in response to nitrogen variation [from sufficiency to starvation (NS to NF stage)] in *H. pluvialis* JNU35.

By using transcriptome sequencing, this study analyzed the expression patterns at eight time-points in the whole culture cycle (from green vegetative cell stage to red cyst cell stage). During the whole culture cycle, the expressions of eight key genes in the MEP pathway were significantly upregulated. The IPP for astaxanthin synthesis probably originated from the MEP pathway. Moreover, all rate-limiting genes involved in astaxanthin synthesis in *H. pluvialis* JNU35 were significantly upregulated, and the other collateral pathways competing with astaxanthin synthesis were suppressed, resulting in the rapid accumulation of astaxanthin under nitrogen starvation.

In the NF stage, the photosynthetic system of *H. pluvialis* JNU35 was attenuated, yet photosynthetic activity was maintained at a very low level. When the Z-type electron-transport chain was affected under the stress, *H. pluvialis* JNU35 may activate the cyclic electron-transport chain containing only PS I as compensation. Accordingly, *H. pluvialis* JNU35 generates the NADPH and ATP by the interaction of the Z-type electron-transport chain and the cyclic electron-transport chain to promote growth and astaxanthin accumulation.

With the reduction of nitrogen concentration, the efficiency of nitrogen absorption was improved. Genes related to nitrogen transport and assimilation, the proteasome pathway, and endocytosis were upregulated. When chloroplasts and nonessential proteins were degraded, the algae could use their own endogenous nitrogen under the nitrogen starvation stress. More significantly, a portion of metabolism was transferred from the chloroplast to the cytoplasm.

## Methods

### Strain and culture conditions

*Haematococcus pluvialis* JNU35 was isolated from a sand pond of YaJiaGeng stream of Gongga Mountain, Sichuan, China. In our laboratory, the pure stock cultures were stored in 250-mL flasks with BBM medium (250 mg L^−1^ NaNO_3_, 75 mg L^−1^ MgSO_4_, 25 mg L^−1^ NaCl, 75 mg L^−1^ K_2_PO_4_, 175 mg L^−1^ KH_2_PO_4_, 25 mg L^−1^ CaCl_2_·2H_2_O, 1.425 mg L^−1^ H_3_BO_3_, 8.82 mg L^−1^ ZnSO_4_·7H_2_O, 1.44 mg·L^−1^ MnCl_2_·4H_2_O, 0.71 mg L^−1^ MoO_3_, 1.57 mg L^−1^ CuSO_4_·5H_2_O, 0.49 mg L^−1^ Co(NO_3_)_2_, 50 mg L^−1^ EDTA·Na_2_, 31 mg L^−1^ KOH, 4.98 mg L^−1^ FeSO_4_·7H_2_O, and 3 μL L^−1^ H_2_SO_4_).

*H. pluvialis* JNU35 was cultivated in bubbling glass column photobioreactors (Ø6 cm × 60 cm) with continuous illumination of 100 μmol m^−2^ s^−1^ (for the green vegetative cell stage) and 400 μmol m^−2^ s^−1^ (for red cyst cell stage) provided by fluorescent light at 25 ± 1 °C and bubbled by compressed air enriching with 1% CO_2_ (v/v) with flow rate (vvm, volume of gas per volume of medium per minute) of 0.2–0.3. To culture *H*. *pluvialis* JNU35 in the above photobioreactors, modified BBM medium (mBBM) containing 9.0 mM urea (18.0 mM nitrogen concentration) (nitrogen sufficient, NS) as a nitrogen source or without urea (0.0 mM of nitrogen concentration) (nitrogen free, NF) was used. After being cultured in NS mBBM for 10 days, *H. pluvialis* JUN35 was transferred into the fresh NF medium for another 10 days of cultivation (NS to NF).

### Growth and photosynthesis characteristics analysis

Biomass concentration was measured based on the method of Gao et al. [52]. A 10.0 mL cell suspension was harvested each day and filtered by a preweighed 0.45-μm nitrocellulose filter membrane (*W*_1_). Subsequently, the membrane with algal biomass was dried in an oven at 105 °C overnight, and then it was removed and placed into a desiccator for 30 min before weighing (*W*_2_). The biomass concentration (DW, g L^−1^) was calculated by the equation

$${\text{DW}} = \left( {W_{ 2} - W_{ 1} } \right) \times 100.$$

A Liquid-Phase Oxygen Measurement System (Hansatech, England) was employed to determine the photosynthetic efficiency, including photosynthetic rate (PR) and respiratory rate (RR). Ten milliliters of culture suspension was collected from a photobioreactor every two days. In this study, to ensure accuracy of the instruments, each culture was diluted to the same cell density (OD750 = 0.5 ± 0.05). Then, the following steps were performed: (1) the culture was placed in a thermostatic water bath in the dark at 25 °C for 30 min; (2) 3 mL of the dark adaption algae cell suspension was placed into a response cup and illuminated at a light intensity of 300 μmol m^−2^ s^−1^ to determine PR; and (3) the light source was turned off to determine RR. The photosynthetic and respiratory rates were expressed as the amounts of oxygen evolution and consumption per milligram in the unit volume cell, respectively, with units of μmol O_2_ mg^−1^ h^−1^.

### Pigment content analysis

Chlorophyll and astaxanthin contents were determined using the methods of Wellburn [53] and Li et al. [54], respectively. Freeze-dried biomass (10 mg) was employed for extraction with 5 mL methanol (for chlorophyll) or dimethyl sulfoxide (DMSO) (for astaxanthin) and maintained in a 70 °C water bath until the cells turned almost colorless. Then, the extraction suspension was centrifuged at 4000 rpm for 5 min to collect the supernatant. The supernatant was diluted to 25 mL with methanol or DMSO, respectively.

The absorbance of chlorophyll was measured using a spectrophotometer (Persee, China) at 665 nm and 652 nm, respectively, and the equation for calculating the content of chlorophylls was as follows: chlorophyll *a* (% DW) = *A*×0.0025 × 100%; chlorophyll *b* (% DW) = *B*×0.0025 × 100% (where *A* and *B* are the chlorophyll concentrations (mg L^−1^), *A* = 16.72 × *A*_652_ − 9.16 × *A*_665_; *B* = 34.09 × *A*_652_ − 15.28 × *A*_665_; and *A*_665_ and *A*_652_ are the absorbance values).

The absorbance of astaxanthin was measured using a spectrophotometer (Persee, China) at 530 nm, and the equation for calculating the content of astaxanthin was as follows: astaxanthin content (% of DW) = 0.025*C*_*A*_ × 100%/*M* (where *C*_*A*_ is the astaxanthin concentration (mg L^−1^), *C*_*A*_ = (*A*_530 _− 0.0107)/0.1556; *A*_530_ is the absorbance value at 530 nm; and *M* is the weight of the algae powder).

### Biochemical composition analysis

One hundred milligrams lyophilized cells was used for total lipid extraction in line with Gao et al. [52] with 2 mL of a 10% DMSO-methanol mixture solution in a 50 °C water bath for 10 min. Subsequently, to collect the supernatant, the mixture was centrifuged at 3000 rpm for 5 min. The residue was re-extracted with 4 mL mixture of diethyl ether and hexane (v:v, 1:1) at 4 °C for 1 h, and then it was centrifuged. The extraction was repeated twice, and the supernatants were pooled together. Finally, distilled water was added to the methanol extract and diethyl ether-hexane extract at a ratio of 1:1:1:1 (water/methanol/diethyl ether/hexane, v/v/v/v). The upper phase with lipids was collected and further dried under N_2_ flow for weighing.

Total protein content was determined as previously described [55]. Twenty milligrams of lyophilized cells was hydrolyzed in 5 mL of 0.5 mM NaOH and maintained at 80 °C in water bath for 30 min. The absorbance of protein sample at 750 nm was measured, and bovine serum albumin served as a standard. The protein concentration of the sample was calculated according to the protein standard curve.

Twenty milligrams of lyophilized cells was employed for analyzing total carbohydrate content [56]. They were hydrolyzed in 5 mL of 0.5 M H_2_SO_4_ at 100 °C water bath for 4 h, followed by the centrifugation at 3500*g* for 10 min. Subsequently, 500 μL supernatant was added and mixed with a 7.5 mL mixture [sulfuric acid (5 mL): H_2_O (1.5 mL): 6% phenol (1 mL)]. Moreover, the absorbance at 490 nm was measured. To quantitate total carbohydrate content, glucose was used to plot a standard curve.

### RNA extraction, library construction, and sequencing

By means of RNAiso Plus (TaKaRa Biotech Co., Beijing, China), total RNA was extracted from *H. pluvialis* JNU35 at different time-points. A total of 5 μg RNA per sample was used as the input material for the RNA sample preparations. Subsequently, different time-point mixed samples and individual samples (2 days, 4 days, 8 days, 10 days, 12 days, 14 days, 16 days, and 20 days) of RNA were employed to select the mRNA. The mRNA was enriched by Oligo(dT) magnetic beads to select poly(A) tails. It was then fragmented into short fragments with fragmentation buffer and reverse-transcribed into cDNA with random primers. Next, the cDNA fragments were purified, and the ends of the fragments were repaired, poly(A) tails were added, and the fragments were ligated to sequencing adapters. The ligation products were selected by agarose gel electrophoresis according to size, and PCR was amplified, and sequenced using an Illumina HiSeq2500 and BGI-500 platform by BGI Biotechnology Co. (Shenzhen, China).

RNA samples were extracted from different culture conditions and different time-points for mixing, and paired-end 150-bp sequencing was performed on the Illumina Hiseq2500 platform (Additional file 1: Table S1). The RNA-seq quantitation data of the eight individual samples was produced by single-end 50-bp sequencing on a BGI-500 platform. A statistical analysis of data is presented in Additional file 1: Table S1. The RNA-seq raw data were deposited to the NCBI Short Read Archive (SRA) under accession number SRP136881.

### Transcriptome assembly and functional annotation

To obtain high-quality clean reads, the raw data containing adaptor sequences, reads with low-quality sequences, and unknown nucleotides were filtered. Transcriptome de novo assembly was performed with the reference transcriptome paired-end reads (2 × 150 bp) using Trinity [57]. Gene function annotation was performed using the BLASTx with an *E*-value threshold of 1*e*−5 with the databases as follows: NR (NCBI nonredundant protein sequences), COG (Clusters of Orthologous Groups of proteins), Swiss-Prot, KEGG (Kyoto Encyclopedia of Genes and Genomes), and GO (Gene Ontology).

### Differential gene expression and gene cluster analysis

The clean reads of the eight individual samples were mapped to the reference transcriptome using Bowtie2 [58]. Gene expression quantitation was estimated by RSEM [59] for each sample. Each unigene was then calculated and normalized to the number of Fragments Per Kilobase Million (FPKM). Based on the expression, the differentially expressed genes (DEGs) of two samples were analyzed using the DEGseq R package. Using a Poisson distribution with false discovery rate (FDR) ≤ 0.001 and fold change ≥ 2 (log2 ratio ≥ 1), the DEGs between the experimental groups (4 days, 8 days, 10 days, 12 days, 14 days, 16 days, and 20 days) and the control group (2 days) were identified by multiple hypothesis testing. The threshold for the significant differential expressions was thus established. Finally, the eight time-point DEGs were clustered with STEM [25] based on OmicShare, a free online platform for data analysis (http://www.omicshare.com/tools).

### Functional classification and pathway reconstruction

According to the analysis of Gene Ontology (GO) and KEGG pathway enrichment, significantly enriched GO terms, metabolic pathways, or signal transduction pathways in DEGs were identified compared to the reference transcriptome background using OmicShare tools. The astaxanthin metabolism pathway of *H. pluvialis* JNU35 was reconstructed by the method of Jaeger et al. [60] based on pathways described for plants and algae [26], *H. pluvialis* [12] and *Chlorella zofingiensis* [3, 61, 62]. For each enzymatic step, the Tbtools [63] were used for heat map, text-manipulation, sequence extraction, and tBLASTx searches against the *H. pluvialis* JNU35 transcriptome. Subsequently, a list of candidate unigenes was compiled.

### NADPH quantitation assays in *H. pluvialis* JNU35

The cell concentrations of NADPH in eight samples (2 days, 4 days, 8 days, 10 days, 12 days, 14 days, 16 days, and 20 days) were measured using the Amplite™ Colorimetric NADP and NADPH Assay Kit (AAT Bioquest, USA) according to the manufacturer’s protocols.

### Quantitative PCR (qPCR) validation

The qPCR primers (Additional file 7: Table S7) were designed using Primer Premier 6.0 software. The qPCR was performed on CFX96 Touch (Bio-rad, California, USA) with PrimeScript™ RT reagent kit and TB Green™ Premix Ex Taq™ II (TaKaRa Biotech Co., Beijing, China) according to the manufacturer’s protocols. Samples were performed in triplicate. The qPCR amplification protocol was 95 °C for 30 s; 40 cycles at 95 °C for 5 s, 60 °C for 30 s, and 72 °C for 30 s, followed by the analysis of melt Curve, and a procedure of 0.5 °C increment at 5 s/step from 65 °C to 95 °C was added. The 2 days sample served as control group. Actin gene was set as reference gene, and the relative expression values were calculated using the 2^−ΔΔCt^ method [64].

## Additional files

**Additional file 1: Table S1.** The raw data generated and mapping on the reference transcriptome.

**Additional file 2: Table S2.** Significantly enriched GO terms in the set of 6 profiles (p-value 0.05).

**Additional file 3: Table S3.** The KEGG pathway of significantly expression pattern.

**Additional file 4: Table S4.** The relevant genes involved in astaxanthin biosynthesis.

**Additional file 5: Table S5.** The relevant genes involved in photosynthesis.

**Additional file 6: Table S6.** The relevant genes involved in nitrogen metabolism.

**Additional file 7: Table S7.** Primers for genes validated by qPCR. **Figure S1.** Comparison of expression patterns by qPCR analyses and transcriptome date.

## Abbreviations

AACT: acetyl-CoA C-acetyltransferase
HMGS: hydroxymethylglutaryl-CoA synthase
HMGR: hydroxymethylglutaryl-CoA reductase (NADPH)
MK: mevalonate kinase
PMK: phosphomevalonate kinase
MDC: diphosphomevalonate decarboxylase
DXS: 1-deoxy-d-xylulose-5-phosphatesynthase
DXR: 1-deoxy-d-xylulose5-phosphate reductoisomerase
MCT: 2-C-methyl-d-erythritol 4-phosphate cytidyltransferase
CMK: 4-diphosphocytidyl-2C-methyl-d-erythritol kinase
MDS: 2-C-methyl-d-erythritol 2,4-cyclodiphosphate synthase
HDS: 4-hydroxy-3-methylbut-2-en-1-yl diphosphate synthase
HDR: 4-hydroxy-3-methylbut-2-enyl diphosphate reductase
IDI: isopentenyl-diphosphate delta-isomerase
FDPS: farnesyl diphosphate synthase
GGPS: geranylgeranyl diphosphate synthase
PSY: phytoene synthase
PDS: phytoene desaturase
ZDS: δ-carotene desaturase
CRTISO: carotene isomerase
LYCb: lycopene beta cyclase
CrtZ: beta-carotene 3-hydroxylase
LYC e: lycopene ε-cyclase
BKT: beta-carotene ketolase
LUT5: beta-ring hydroxylase
LUT1: carotene epsilon-monooxygenase
VDE: violaxanthin de-epoxidase
ZEP: zeaxanthin epoxidase
CBCCD: 9-cis-beta-carotene 9′,10′-cleaving dioxygenase
CarS: carlactone synthase
TPS: linalool synthase
DHDDS: ditrans, polycis-polyprenyl diphosphate synthase
FDFT: farnesyl-diphosphate farnesyltransferase
FNTB: protein farnesyltransferase subunit beta
GGPPR: geranylgeranyl diphosphate
SPS: all-trans-nonaprenyl-diphosphate synthase
GES: geranyllinalool synthase
