# Supplementary material for: Comparative transcriptome analysis of a long-time span two-step culture process reveals a potential mechanism for astaxanthin and biomass hyper-accumulation in Haematococcus pluvialis JNU35
Source: Biotechnol Biofuels. 2019 Jan 28;12:18. doi: 10.1186/s13068-019-1355-5 (PMC6348685; doi:10.1186/s13068-019-1355-5)
Supplement: Supplementary file 1 — Additional file 1: Table S1. The raw data generated and mapping on the reference transcriptome. [file 13068_2019_1355_MOESM1_ESM.docx]

**Table S1** The raw data generated and mapping on the reference transcriptome

| **Sample** | **RNA-seq**  **Strategy** | **Clean Bases (Gb)** | **Clean Reads (Mb)** | **Clean ReadsRatio(%)** | **Q20 (%)** | **Mapped Reads (%)** | **Unique Match (%)** | **Multi-position Match (%)** | **Unmapped Reads (%)** |
| --- | --- | --- | --- | --- | --- | --- | --- | --- | --- |
| Reference transcriptome | PE150 | 11.13 | 74.19 | 94.83 | 97.15 | NA | NA | NA | NA |
| 2d | SE50 | 2.3 | 47.14 | 99.92 | 96.7 | 88.67 | 52.59 | 36.08 | 11.33 |
| 4d | SE50 | 2.54 | 52.02 | 99.8 | 96.6 | 88.87 | 51.77 | 37.1 | 11.14 |
| 8d | SE50 | 2.93 | 60.1 | 99.77 | 96.4 | 88.35 | 50.78 | 37.57 | 11.65 |
| 10d | SE50 | 2.53 | 51.82 | 99.92 | 96.8 | 88.63 | 51.77 | 36.86 | 11.37 |
| 12d | SE50 | 2.34 | 47.91 | 99.85 | 92.9 | 86.16 | 46.01 | 40.15 | 13.85 |
| 14d | SE50 | 2.68 | 54.8 | 99.45 | 94 | 86.71 | 47.12 | 39.59 | 13.29 |
| 16d | SE50 | 2.25 | 46.14 | 99.77 | 94.3 | 87.35 | 47.86 | 39.49 | 12.65 |
| 20d | SE50 | 2.89 | 59.29 | 99.91 | 93.4 | 87.27 | 47.36 | 39.91 | 12.73 |
