# Supplementary material for: Comparative transcriptome analysis of a long-time span two-step culture process reveals a potential mechanism for astaxanthin and biomass hyper-accumulation in Haematococcus pluvialis JNU35
Source: Biotechnol Biofuels. 2019 Jan 28;12:18. doi: 10.1186/s13068-019-1355-5 (PMC6348685; doi:10.1186/s13068-019-1355-5)
Supplement: Supplementary file 7 — Additional file 7: Table S7. Primers for genes validated by qPCR. Figure S1. Comparison of expression patterns by qPCR analyses and transcriptome date. [file 13068_2019_1355_MOESM7_ESM.pdf]

Table S7 Primers for genes validated by Quantitative real-time PCR (qPCR)

Genes	Unigene ID	Primer	Sequence (5′–3′)	Product size (bp)
DXS	Unigene7019_A	DXS -F	TAAGACGGGCGGCCACTTGA	112
		DXS -R	CTGGTGGCCCACATCCCAGA	
HDR	CL6996.Contig1_A	HDR-F	TCCCTAGGCAGCACCACCCT	167
		HDR-R	GGCCTGAAGACGCGTAGCGA	
FDPS	Unigene23365	FDPS -F	CTCCAAGCCTGATGCCTAGATTGC	116
		FDPS -R	CCACACTGAGCCAGCGAATAGC	
GGPS	Unigene30715_A	GGPS -F	CCTCATGGAATCAACCAGCACCTC	159
		GGPS -R	GTCTCAGCCGTAGCAACAGCAG	
PSY	CL4515.Contig1_A	PSY-F	TGCCACTCTCCACCTATTCCACTC	199
		PSY-R	CCAGCAGCAGGCGACTTGTATG	
CrtZ	Unigene20243_A	CrtZ-F	GGCGAGATGCCTTGGAGCGA	165
		CrtZ-R	AAAAGGTCCGGTGCGGGGAG	
BKT1	Unigene11485_A	BKT1-F	CTGTCCGCCTTCCGCTTGTTC	161
		BKT1-R	AGGTCGAAGTGGTAGCAGGTCAG	
BKT2	CL7868.Contig1_A	BKT2-F	AGGTTGGGCAGAGCACGGAG	148
		BKT2-R	AGTTCACGCGGGGCAACTCA	
actin	CL9171.Contig4_A	Actin-F	ACACCCGAGATCCCCACGGT	166
		Actin-R	AGCGAACCCGGCTTTCACCA	

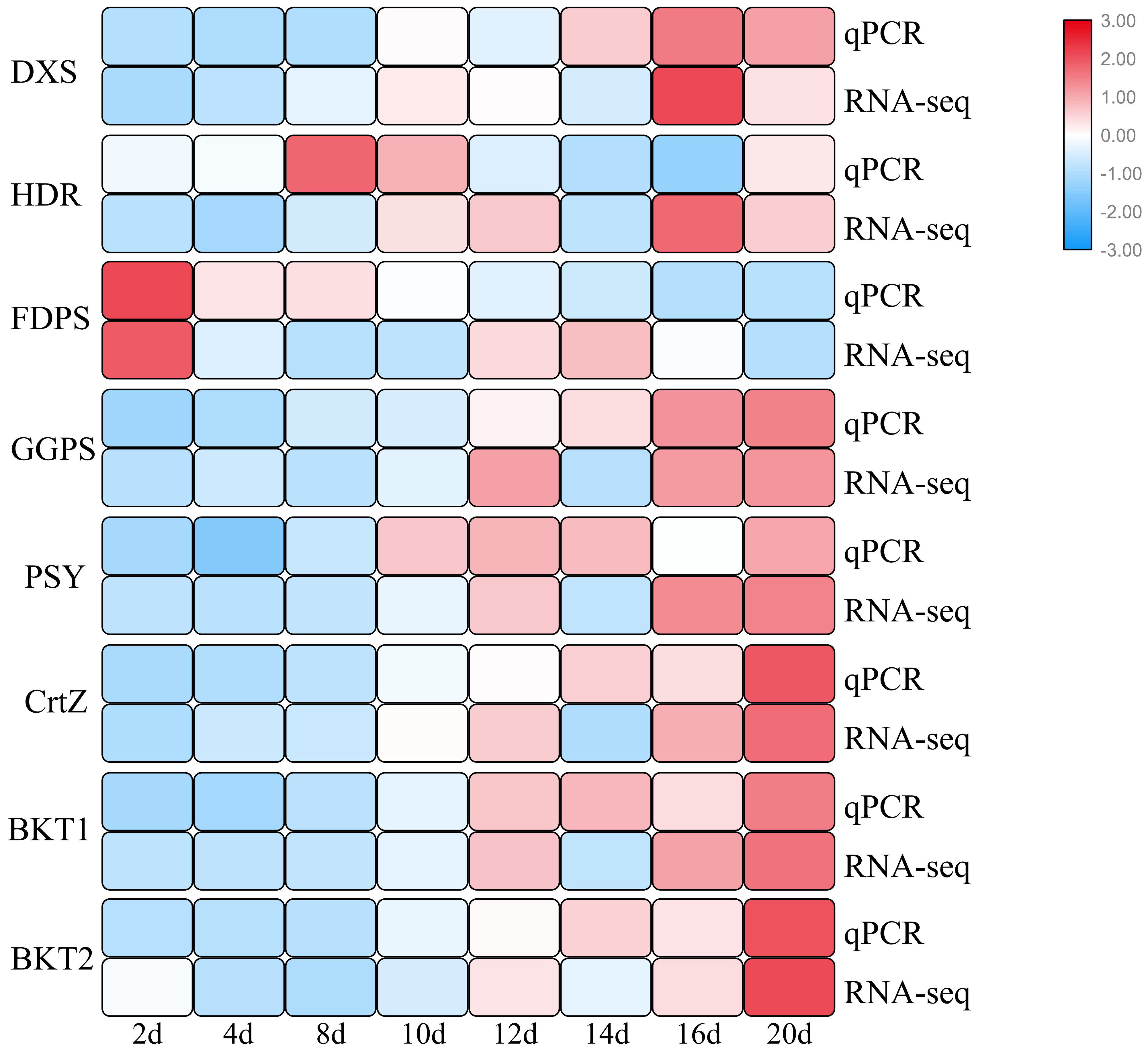
DXS
HDR
FDPS
GGPS
PSY
CrtZ
BKT1
BKT2
qPCR
RNA-seq
2d
4d
8d
10d
12d
14d
16d
20d
3.00
2.00
1.00
0.00
-1.00
-2.00
-3.00
